# Progress in Polymeric Micelles for Drug Delivery Applications

**DOI:** 10.3390/pharmaceutics14081636

**Published:** 2022-08-05

**Authors:** Sabna Kotta, Hibah Mubarak Aldawsari, Shaimaa M. Badr-Eldin, Anroop B. Nair, Kamal YT

**Affiliations:** 1 Department of Pharmaceutics, Faculty of Pharmacy, King Abdulaziz University, Jeddah 21589, Saudi Arabia; 2 Center of Excellence for Drug Research and Pharmaceutical Industries, King Abdulaziz University, Jeddah 21589, Saudi Arabia; 3 Department of Pharmaceutical Sciences, College of Clinical Pharmacy, King Faisal University, Al-Ahsa 31982, Saudi Arabia; 4 Department of Pharmacognosy, College of Pharmacy, King Khalid University, Abha 61421, Saudi Arabia

**Keywords:** critical micelle concentration, drug delivery, oral, polymeric micelles, tumor-targeted

## Abstract

Polymeric micelles (PMs) have made significant progress in drug delivery applications. A robust core–shell structure, kinetic stability and the inherent ability to solubilize hydrophobic drugs are the highlights of PMs. This review presents the recent advances and understandings of PMs with a focus on the latest drug delivery applications. The types, methods of preparation and characterization of PMs are described along with their applications in oral, parenteral, transdermal, intranasal and other drug delivery systems. The applications of PMs for tumor-targeted delivery have been provided special attention. The safety, quality and stability of PMs in relation to drug delivery are also provided. In addition, advanced polymeric systems and special PMs are also reviewed. The in vitro and in vivo stability assessment of PMs and recent understandings in this area are provided. The patented PMs and clinical trials on PMs for drug delivery applications are considered indicators of their tremendous future applications. Overall, PMs can help overcome many unresolved issues in drug delivery.

## 1. Introduction

The emergence and tremendous progress of polymer-based drug delivery carriers and systems have surpassed the conventional medication approaches. Although diffusion-controlled systems were popular earlier with the use of polymers, intelligent drug delivery systems are now exploited. Site-specific targeting, the enhancement of bioavailability and stimuli-sensitive release and feedback strategies are some of the most sought advantages of intelligent systems using polymer-based drug delivery. When biocompatibility posed some objections to the extensive use of polymers in drug delivery, a plethora of biocompatible polymers emerged. Moreover, chemical engineering enabled the emergence of new polymers and modifications of existing polymers to suit intelligent systems [1,2,3].

Simultaneously, the drug delivery system witnessed the parallel and exponential growth of polymer nanotechnology. Polymeric nanostructures are able to achieve bioavailability and site-specific delivery. Furthermore, polymers are able to provide desirable particle size, surface properties, permeation profiles and flexibility to nanostructures. These advantages are in addition to the basic requirements of enhanced solubility and controlled drug release. The possibilities of bioengineering and functionalization further strengthen the utility and applications of polymeric nanostructures. All these have led to their wide applications in biomedicine. Nanospheres, nanocapsules, nanocomposites, nanofibers, dendrimers, nanogels, polymersomes and polymeric micelles (PMs) are the major polymeric nanostructures for drug delivery applications [4,5,6,7].

The presence of a robust core–shell structure, kinetic stability and their inherent ability to solubilize hydrophobic drugs are considered advantages of PMs. Recently, PMs have been explored for a variety of drug-delivery applications. The variety and versatility of polymers available for the fabrication of PMs enhance their opportunities in drug-delivery applications. In addition to the core-forming and corona-forming polymers, polymers that can impart stimuli-responsive nature to PMs have also been reported [8]. The specific application of PMs for drug delivery depends on their types. Moreover, the method of their preparation can influence parameters such as drug loading and entrapment efficiency. These factors can influence the safety, quality and stability of PMs, which in turn influence their extent of application [7,9,10].

## 2. Types of Polymeric Micelles

Depending on the polymer and solution attributes, several possibilities exist in the formation of different types of PMs. The type of polymer used influences the self-assembling process to form PMs. Thus, di-block, tri-block, multi-block copolymers, graft polymers, stimuli-sensitive polymers, etc., can produce different PMs. Moreover, the solvent, pH, polymer concentration and ratios, co-solvent, etc., can significantly influence the type that is formed. The term PMs is generally applied to systems where the hydrophilic part of the amphiphilic polymer is directed outwards, and the lipophilic part is directed to the core of the micelles. In the case of reverse micelles, the hydrophilic part of the amphiphilic polymer is directed towards the core, and the lipophilic part is directed outwards. Mixed micelles are prepared by the inclusion of solubilizates into surfactant micelle. With the addition of surfactants, the lipid bilayer dissolves and results in the formation of mixed micelles made up of both surfactants and polar lipids [11].

PMs are made from mainly diblock polymers, triblock polymers or graft polymers with hydrophilic and hydrophobic portions. They can be made up of ionic copolymers with ionic and hydrophilic parts. Based on the intermolecular forces controlling the segregation of micelles in aqueous surroundings, block copolymers are divided into three types. They are amphiphilic micelles (hydrophobic interactions), polyion complex micelles (electrostatic interactions) and micelles formed by metal complexations [12]. Based on the mode of the encapsulation of drugs, PMs are mainly classified into two, either by chemical covalent binding of drugs or the physical encapsulation method. The drug can be encapsulated in different regions of the PMs according to the polarity. Nonpolar drugs on the core, polar drugs on the shell and drugs with intermediate polarity encapsulated between the core and the shell [13].

Polymeric micelles consist of several compartments on their hydrophobic cores and are surrounded by a single hydrophilic shell. Based on the micellar architecture, micelles are of different types, viz, regular, snow man shape, two hemisphere shape, cylindrical, reverse shape, dumbbell shape, etc. Multicompartment systems also show different shapes, such as disc-like, worm-like, raspberry-like, sheet-like, etc. [12].

## 3. Methods of Preparation

The method of preparation for PMs is based on the physicochemical characteristics of the block copolymers selected for the PM preparation. The chosen method significantly affects the physicochemical parameters and drug encapsulation efficiency. The order of addition, the ratio of aqueous/organic ratio and the concentration of the copolymers affect the size, poly dispersity index and stability. Therefore, optimizing these parameters is advantageous to obtain a standard formula for making PMs with good physicochemical and functional characteristics. Different types of polymers used for the micelle fabrication and their methods of preparation are shown in Table 1.

### 3.1. Direct Dissolution Method

Copolymers with high aqueous solubility are mainly used for making PMs by the direct dissolution method. This method is relatively easy and simple and involves the mixing of copolymers and drugs in aqueous solvents in the presence of mechanical methods such as stirring, sonication and heating to encapsulate the drugs. The dehydration of the core-forming blocks leads to the formation of PMs. The copolymers and drugs are dissolved separately in aqueous solvents and mixed to obtain PMs [41,42].

### 3.2. Simple Mixing

In this method, PMs are constructed based on the self-assembly of oppositely charged block copolymers in an aqueous environment. Following this multivalent electrostatic complexation, the charged macromolecules such as nucleic acids, proteins and oligonucleotides are encapsulated in the core compartment, and the PEG chains surrounding the core form the protective shell compartment. This simple method does not require dialysis, solvent evaporation or microfluidics. This method is widely used for preparing PMs assembled via electrostatic interactions, which are known as polyion complex (PIC) micelles of positively charged PEG-b-poly(L-lysine) and negatively charged PEG-b-poly(aspartic acid). However, PIC micelles produced by this method may be fragile under physiological conditions and may disassociate and separate as a result of charge shielding by salts and interactions with naturally occurring polyelectrolytes or charged proteins. Hence, PIC micelles must be stabilized to make them beneficial under biological conditions [43].

### 3.3. Solvent Evaporation Method

The solvent evaporation method involves the dissolution of both copolymers and drugs in a common solvent or miscible solvents. When both the polymers are dissolved in the solvent and are soluble in the water, this is the method of choice for PM fabrication. A drug-copolymer thin film is formed after the evaporation of the solvents. The addition of water or buffers leads to the spontaneous formation of drug-loaded PMs. Furthermore, processing by a sonicator or high-pressure extruder distributes the size of the micelles equally [42,44].

### 3.4. Dialysis Method

The dialysis method is used when the selected amphiphilic copolymers have low water solubility. The copolymer and drug are dissolved in a common solvent, followed by the addition of an aqueous solvent to stimulate the micelle formation. The mixture is dialyzed against water for a long period to remove the organic solvents. The selection of solvent is essential for this method, as it affects the physical characteristics of the micelles and drug encapsulation efficiency. An optimum ratio of aqueous and organic solvent is also very important. Commonly used solvents for this method are *N*,*N*-dimethylformamide, dimethylsulfoxide, acetone, acetonitrile, tetrahydrofuran, etc. [42,44].

### 3.5. Continuous Processing

**Figure 1:**
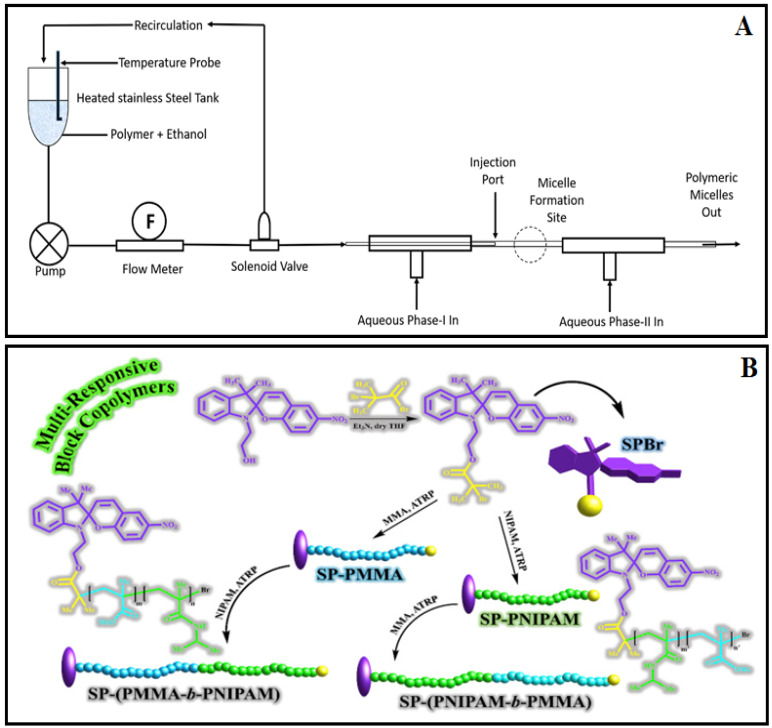
(**A**) Schematic representation of continuous processing method for micelle formation. Reprinted with permission from [14] © Elsevier 2020. (**B**) Schematic representation of the design of multi-responsive block copolymers having spirogyra end groups by atom transfer polymerization method. Reprinted with permission from [47] © Elsevier 2019.

A co-axial turbulent jet with co-flow continuous technology has been recently introduced for producing PMs [14]. This type of system has been previously reported for liposome preparation [45]. Continuous processing was possible with this system. A diagrammatic sketch of the setup is shown in Figure 1A. In the demonstration of this method, a block copolymer of mPEG (5 kD)-PCL was used as the polymer for loading curcumin. The method was found to produce micelles with high drug loading and low polydispersity indices. The control over the processing parameters is another advantage of this method over the conventional preparation method [14].

### 3.6. In Situ Charge-Neutralization-Controlled Particle Coagulation Mechanism

Recently, monodisperse polymeric particles have been prepared by an in situ charge-neutralization-controlled particle coagulation mechanism. Cationic radicals formed from the initiator decomposition shield the anionic surfactant molecules adsorbed on the surface of particles and support the particle coagulation. The number of particles decreases, and the rate of micelle capture for initiator radicals is enhanced with in situ charge neutralization. Thus, the particle nucleation period shortens, and monodisperse particles of 200–300 nm size form with this simple emulsion polymerization process [46].

### 3.7. Spiropyran-Initiated Atom Transfer Polymerization

Spiropyran-initiated atom transfer polymerization is another recent technique used to prepare light and temperature-sensitive PMs. Spiropyran is one of the most applicable photochromic compounds having high light responsivity, high sensitivity, great photo fatigue-resistant characteristics and reversibility. Various types of multi-responsive PMs consist of spirogyra chain end groups, temperature-sensitive poly(N-isopropyl acrylamide)(PNIPAM) block and poly (methyl methacrylate)(PMMA), which were prepared by this method (Figure 1B) [47].

## 4. Methods of Characterization

In order to understand and predict the activities of micelles in a biological environment, characterization is an important task. The characterization of the formed micelles demands the collaboration of various techniques and approaches. The chemical composition of the block copolymers greatly impacts the polymer self-association, physicochemical characteristics and in vitro and in vivo behaviors. The importance of proper characterization is mentioned in the Joint MHLW/EMA reflection paper on the development of five-block copolymer micelle medicinal products [48].

### 4.1. Critical Micelle Concentration Determination (CMC)

CMC determination is an important part of PM characterization. It mainly represents the hydrophobic and hydrophilic segment balance. Characteristics of hydrophobic groups, the molecular weight of the hydrophilic part and the distribution of the hydrophilic part in the amphiphilic polymer influence the CMC [49]. Many methods are available for the determination of CMC. Light scattering, surface tension and electrical conductivity are reporter-free methods for CMC determination that are based on changes in macroscopic parameters. Photometric and fluorometric methods are other common methods for CMC determination with the aid of suitable optical probes [50].

The determination of CMC by surface tension can be performed by the Wilhelmy plate method [51]. When the concentration of amphiphiles increases, the surface tension decreases until the CMC comes to a constant value. Surface tension is almost constant at a concentration of the polymer above the CMC value. Even though the sample preparation method is easy, this technique requires more time and a large number of samples [52].

The fluorescence or absorbance technique can be used for the determination of CMC by measuring the signal exhibited by a dye in the micelles with higher concentrations of amphiphilic polymers. The CMC can be determined by this method via the commonly used pyrene method. Pyrene is a hydrophobic fluorescent molecule and can be used as a fluorescent probe for CMC determination. Pyrene causes a shift of excitation wavelength due to partitioning in the hydrophobic cores of the PMs [49,53]. Other agents used for this techniques are Nile red [54], Sudan-III [55], 1,6-diphenyl-1,3,5-hexatriene [56], etc. CMC values of the copolymer can also be determined by the well-reported iodine method [57].

Dynamic light scattering (DLS) is another technique for the determination of CMC. The scattered light in the DLS method is based on the molecular weight of the particles in micellar solutions. The intensity of light scattering shows a constant value below the CMC, but it increases markedly when the concentration reaches CMC [58]. DLS is a rapid, high-throughput method for CMC determination. However, this technique is not suitable for the determination of samples with low polymer concentrations (below 50 ppm) [52,58].

### 4.2. Morphological Characterization

Various microscopic techniques can be used for the morphological characterization of PMs. There are various microscopic imaging techniques for micelle characterization. AFM is a high-resolution microscopical technique that is useful for the analysis of the morphology and size of the micelles. It can also be used for the evaluation of redox or temperature-related morphological changes. The tapping mode of AFM is particularly preferred for micelles to reduce the distortion of the micellar structure while analyzing. The AFM method is based on the deposition of the micelles on a solid support. The deposited sample is analyzed by a sharp probe, and its movement is based on the topography of the fixed sample.

Cryo-TEM is another powerful tool used to determine the morphology of micelles. Compared to normal TEM, cryo-TEM allows the evaluation of micelles in their solution state. As the liquid background is important for micelles, cryo-TEM is preferred for micelle characterization. However, this technique is highly time-consuming and expensive [59]. SEM is also used for the analysis of morphological characters [60].

High-resolution imaging techniques fail to capture the ‘snapshots’ of frozen soft nanomaterial, hence providing inadequate temporal resolution. These techniques do not provide any data on a particle-by-particle basis in their native environment. However, Liquid Cell Transmission Electron Microscopy (LC-TEM) can visualize the self-assembly and progression of block copolymers in real time. It can help to learn the theory of micelle formation, the organization of copolymers and morphological characterization [61].

### 4.3. Physicochemical Characterization

**Figure 2:**
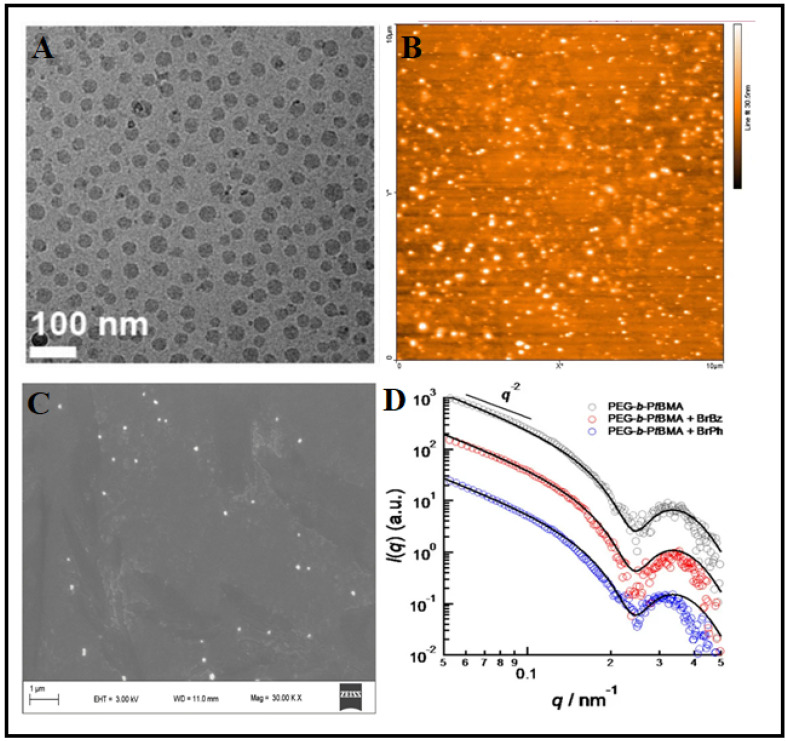
Various characterization techniques for PMs: (**A**) Cryo-TEM images surfactant copolymer complex micelle. Reprinted with permission from [71]. (**B**) AFM image of self-assembled cholesterol end-capped polymeric micelle. Reprinted with permission from [49]. (**C**) SEM image of self-assembled cholesterol end-capped polymeric micelle. Reprinted with permission from [49]. (**D**) SAXS profile of bromobenzene (BrBz) and 4-bromophenol (BrPh) encapsulated PMs made up of poly(ethylene glycol)-*b*-poly(*tert*-butyl methacrylate) (PEG-*b*-PtBMA). Reprinted with permission from [72].

Structural aspects of the synthesized copolymer can be analyzed by employing Fourier Transform Infrared Spectroscopy (FTIR) and Nuclear Magnetic Resonance Spectroscopy (NMR) spectroscopy [35,62] Small-angle Neutron Scattering (SANS) and Small Angle X-ray Scattering (SAXS) are two powerful tools used for the structural analysis of micelles. With these techniques, it is possible to find the hydrophobic and hydrophilic contributions of a micelle [63]. Anomalous Small-Angle X-ray Scattering (ASAXS) is another valuable tool used to determine the spatial distribution of hydrophobic molecules in polymeric micelles [64]. The thermal behavior of polymeric micelles can be determined by Differential Scanning Colorimetry (DSC) [65]. Figure 2 represents the characterization of micelles with various techniques.

### 4.4. Monitoring the Nano-Bio Interaction

Various tools have been developed to understand the physicochemical interactions between PMs and biological systems. For example, confocal laser scanning microscopy (CLSM) has been used to study the subcellular trafficking and endosomal escape capability of PIC micelles nucleic acids through endocytosis. Miyamoto et al. used CLSM to observe the internalization of the peptide-modified micelles into plant cells. Moreover, subcellular localization and the colocalization of the micelles in epidermal cells have been observed [66]. Similarly, intravital CLSM has been used to monitor the various journey events of nanocarriers, including PMs in the body [67] and including extravasation into tumors [68,69], blood circulation profiles, capturing and the fate of PIC micelles by liver sinusoidal wall cells [70].

## 5. Molecular Dynamics of Polymeric Micelles

An interesting study on the effect of drug loading in PMs has recently been reported. In this study, the micelles were prepared using 1,2-distearoyl-*sn*-glycero-3-phosphoethanolamine-*N*-mPEG (2 kDa) polymer and the effect of the loading of the paclitaxel was studied. Both the physical and biological effects of drug loading were evaluated. The micelle size was characterized with respect to the average size, core size and shell radius. The average size of the micelles was found to be the least affected by the drug incorporation. Nevertheless, the small-angle X-ray scattering results showed that an increase in core size happens on the loading of paclitaxel. Moreover, this increase in core size is compensated by a reduction in shell radius. These two events finally result in an unchanged micelle size when analyzed by dynamic light scattering. The in vivo evaluation in mice using ^99m^Tc radiolabeled-polymeric micelles showed that the blank micelles were having longer circulation time than paclitaxel-loaded micelles. Conformation changes in polyethylene glycol (PEG) polymers have been described to influence the plasma half-life of micelles. The brush conformation changed to a mushroom conformation on paclitaxel loading [18].

Increasing the concentration of surfactants in polymer-surfactant systems can damage the structure of surfactant micelles. This is expected to influence the polymer conformation in the dilute regime (polymer chains are singles) and in the entangled regime (polymer system is described by the blob concept). The effect of a surfactant sodium bis(2-ethylhexyl) sulfosuccinate on the conformation of different molecular weight polyvinylpyrrolidone (PVP) in a dilute regime showed morphological transitions with increasing concentrations. The surfactant addition led to the expansion of PVP chains. The entangled regime addition of surfactant did not affect the pseudoplastic or Newtonian behavior of PVP [73].

Researchers have examined the morphological properties of block copolymer micelles confined in noncylindrical geometrical structures in a study. The combination of nonporous materials and polymeric systems facilitates applications such as nanolithography and catalysis. The morphology of polystyrene block copolymer micelles confined in anodic aluminium oxide template nanopores is affected by metal precursors, chloride trihydrate and annealing solvents. The selective ion complexation of chloride trihydrate and polystyrene block copolymers and the effective tuning of annealing solvent vapors can control the morphology of micelles [74].

**Figure 3:**
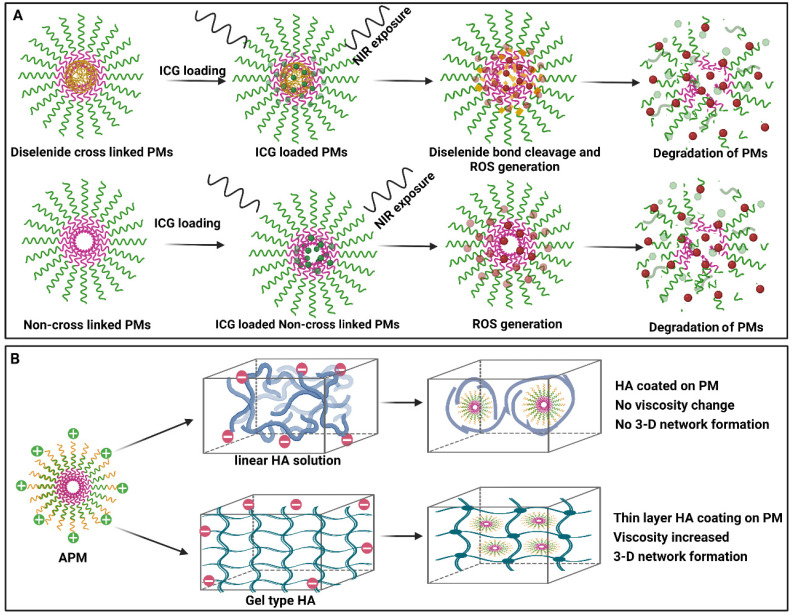
(**A**) Effect of exposure of NIR light on core cross-linked and non-core cross-linked polymeric micelles. (**B**) Application of electrostatically attractive PMs on the rheology of HA gel and solutions.

The effect of the exposure of near-IR light on the PMs formed with a block copolymer has been reported. The micelles were prepared with poly(ethylene glycol)-block-poly(styrene-alt-maleic anhydride) polymers. Diselenide core cross-linked and non-cross-linked PMs assisted with indocyanine green were employed in the study. It was observed that, on exposure to near-IR light, the indocyanine green (ICG) produces reactive oxygen species, and it causes the degradation of the micellar system. However, the study showed that the NIR exposure not only supports the diselenide bond cleavage, but it causes the degradation of PEG chains. Therefore, the poly(ethylene glycol) group in the polymer becomes degraded on exposure to near-IR light and thus results in the continuous destruction of the micelles for 36 h even after NIR exposure stops (Figure 3A) [75].

The polymer block length of poly(ethylene glycol)-block-poly(ε-caprolactone) (PEG-*b*-PCL) influences many of the drug delivery aspects of the PMs. Low-molecular-weight polymers favor permeation and cellular uptake, whereas high-molecular-weight polymers enhance the solubilization of drugs. These results were observed when paclitaxel was used as the drug [76].

The application of PMs in controlling the rheology of hyaluronic acid solutions has been demonstrated. In this case, poly(2-aminoethyl methacrylate)-block-poly(ε-caprolactone)-block-poly(2-aminoethyl methacrylate) polymer was co-assembled with poly(ethylene oxide)-block-poly(ε-caprolactone) to make electrostatically attractive polymeric micelleS (APMs). The electrostatic interactions between the oppositely charged polymeric micelles and hyaluronic acid polymer chains result in changes in the rheology of the system. The positively charged PMs act as particulate cross-linkers. These modifications can be used in controlling the rheology of hyaluronic acid hydrogel for varied drug delivery applications (Figure 3B) [77].

## 6. Stability of Polymeric Micelles

Drug diffusion and the disassembly of micelles are the methods of drug release from PMs. Various characteristics lead to inadequate or poor stability of micelles in blood. Micelles should possess adequate kinetic and thermodynamic stability to avoid uncontrolled drug release during administration. There are certain methods to increase the stability of micelles, such as reductions in CMC, the functionalization of micelles with hydrophobic blocks, core cross-linking methods, the usage of conjugated block copolymers, etc. Serum proteins are important factors for considering the stability of micelles in vivo, since they can form protein coronas over the surfaces of the micelles. Therefore, micellar stability investigations in bio-relevant conditions are very important [52].

The thermodynamic stability of micelles is important in drug delivery applications. A free-energy-based evaluation of the thermodynamic stability of ibuprofen-loaded poloxamer micelles was carried out in a study. It was observed that the free energy was inversely related to the quantities of the drug and polymer present in the micelles. The free energy increased when the amount of free drug and polymer increased in the medium [78].

## 7. Safety and Quality of Polymeric Micelles

The optimization or balancing of anti-cancer activity and the toxicity of drug-loaded PMs were demonstrated in a study. Cisplatin and poly (L-glutamic acid)-*g*-mPEG (5 KDa) were used as the drug and the polymer, respectively. The micelles were formed by self-assembly of the polymer. The co-existence of single and double complexation was observed in the cisplatin-loaded PMs. A cisplatin:copolymer ratio of 1:3 provided high therapeutic activity with low toxicity. The fixed aqueous layer thickness and PEG density can influence the in vivo fate and circulation time of the PMs. At a cisplatin:copolymer ratio of 1:3, the PEG density and fixed aqueous layer thickness are high at the surface of the micelles and provide better biocompatibility and therapeutic effects [79]. Poloxamer 407 has been found to show a reduction in the hemolytic potential of amphiphilic drugs [80].

## 8. Drug Delivery Applications

One of the main challenges in the clinical translation of PMs is the retention of the drug in the nanocarrier system upon its systemic administration [15].

### 8.1. Oral

**Figure 4:**
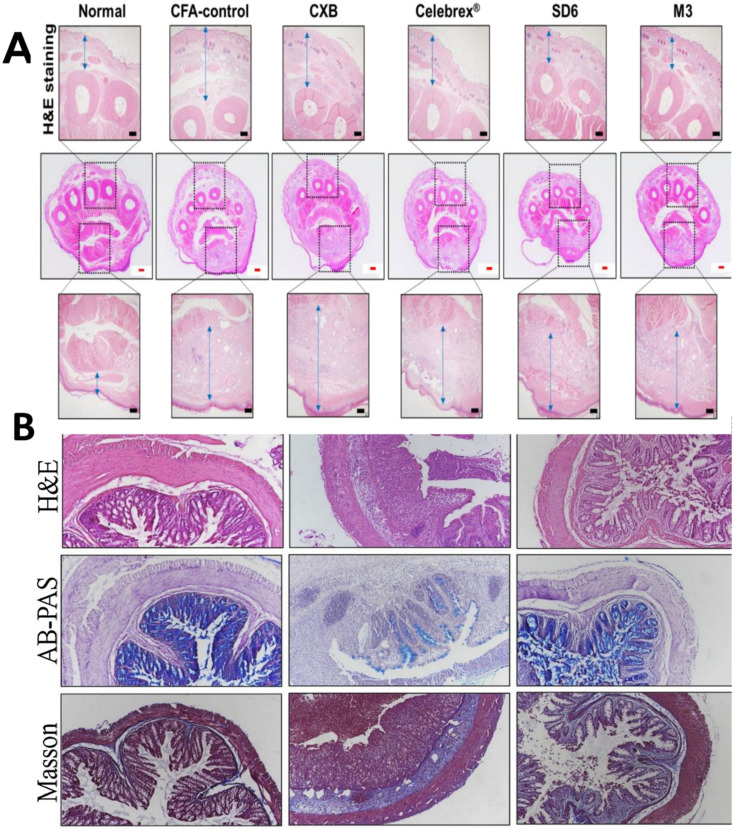
(**A**) Histological micrographs (10× and 40× magnification) of paw tissues of normal, complete Freund’s adjuvant (CFA) control, Celecoxib (CXB), Celebrex^®^, previously developed solid dispersion (SD6) and optimal CXB micelles (M3) in CFA-induced rats with H&E staining. Reproduced with permission from [81] © 2020 Elsevier. (**B**) Hematoxylin-Eosin (HE), Alcian Blue Periodic acid Schiff (AB-PAS) and Masson staining results of the colons of colitis mice after treatment with different formulations (20× magnification). Reproduced with permission from [26] © 2020 Elsevier.

A reported oral drug delivery system of celecoxib uses micelles formed with Soluplus^®^ (polyvinyl caprolactam–polyvinyl acetate–polyethylene glycol graft copolymer) and Kolliphor^®^ HS-15 (macrogol (15)-hydroxy stearate) polymers. The solvent evaporation method was used for the preparation of micelles. The PMs were found to enhance the bioavailability and in vivo anti-inflammatory activity in rats when compared to a marketed formulation. Nevertheless, these effects were lower when compared to a developed solid dispersion formulation of celecoxib. Thus, further fine-tuning is required to enhance the performance of PMs for celecoxib (Figure 4A) [81].

Amphiphilic chitosan micelles can be formed by modifying chitosan with appropriate chemical groups. In a study, the grafting of O-methyl-O′-succinyl PEG and oleic acid to chitosan provided amphiphilic micelles with a structural composition of mPEG-chitosan-oleic acid. The critical micelle concentration of the system was found to be around 0.07 to 0.150 mg/mL. Camptothecin-loaded PMs were prepared using the dialysis method. The micelles showed an entrapment efficiency of 78% and a camptothecin loading of 5%. The systems demonstrated a sustained in vitro release of camptothecin. Furthermore, the micelles protected camptothecin from hydrolysis in the simulated gastrointestinal fluid, thereby providing a potential oral drug delivery platform [82].

### 8.2. Parenteral

An injectable polymeric micelle formulation of laquinimod uses D-α-tocopherol PEG 1000 succinate (TPGS) as the micelle-forming polymer. The formulation was intended for inflammatory bowel disease. A drug:polymer ratio of 1:60 was employed in the optimized formulation. The formulation prepared by thin-film dispersion had a good entrapment efficiency of 89.3  ±  1.0%. The mean particle size and zeta potential of the prepared micelles were found to be 34.6  ±  0.8 nm and −0.67  ±  0.55 mV, respectively. The encapsulation of laquinimod in TPGS micelles aided in enhancing its solubility to 500 μg/mL and facilitated the injectable formulation of this drug. Furthermore, the laquinimod-loaded micelles were found to be effective against induced colitis in mice. The laquinimod-loaded micelles alleviated the severity, facilitated recovery and reduced the inflammation in the in vivo studies in mice (Figure 4B) [26].

**Figure 5:**
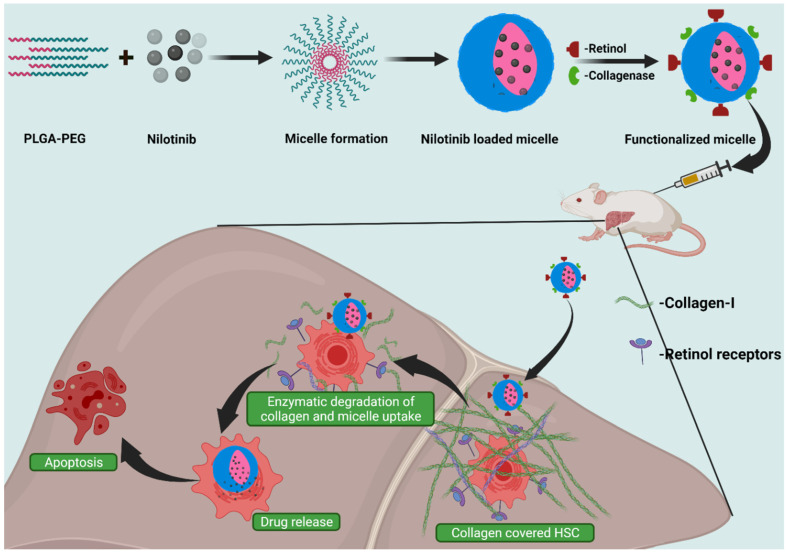
Design and mechanism of the action of nilotinib-loaded functionalized micelles for liver fibrosis.

When collagenase I and retinol are co-attached to micelles formed with poly-(lactic-co-glycolic)-b-poly (ethylene glycol)-maleimide, an efficient drug delivery vehicle to hepatic stellate cells is obtained. The rationale for the use of retinol can be understood by the fact that hepatic stellate cells are storage centers for retinol and have surface receptors for retinol. The use of collagenase I is for the digestion of collagen I deposited during liver fibrosis. The deposited collagen I can impede drug delivery to hepatic stellate cells. Thus, the use of the co-attachment of collagenase I and retinol enhances the targeting and ease of a therapeutic agent delivery into the hepatic stellate cells. When nilotinib is delivered using this system, an optimum activity is observed against liver fibrosis (Figure 5) [38].

In addition to targeting tumor cells, PMs could also be used for targeting tumor-associated macrophages. Such immunotherapy would be beneficial for drugs such as imiquimod. The intratumoral administration of imiquimod-loaded PMs was tried along with the intravenous administration of doxorubicin-loaded PMs for chemo-immunotherapy. The micelles were prepared with phenylboronic acid-poly(ethylene glycol)-poly(ε-caprolactone) and acetylated chondroitin sulfate-protoporphyrin polymers for the preparation of doxorubicin-loaded and imiquimod-loaded PMs, respectively. Both the PMs were prepared by the solvent evaporation method. A high tumor inhibition rate (85%) and the high survival rate of mice (80%) were observed with this synergistic system. Thus, this synergistic therapy can prove to be a promising strategy [83].

### 8.3. Antibacterial

**Figure 6:**
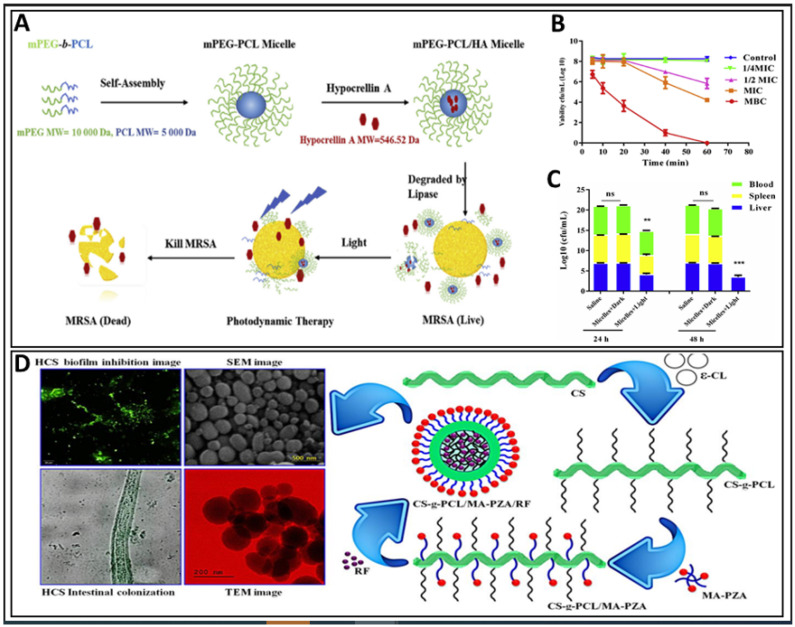
(**A**) Preparation of (mPEG-b-PCL) HA micelle for photodynamic antibacterial activity. (**B**) Antibacterial activity of micelles at different irradiation times. (**C**) Total bacterial count in blood, spleen and liver after dark and light irradiation with micelles for 24 and 48 h. Reprinted with permission from [22] © Elsevier 2019. ** *p* < 0.01, *** *p* < 0.001. (**D**) Schematic representation of fabrication, characterization and evaluation of CS-g-PCL/MA–PZA micelles. Reprinted with permission from [84] © Elsevier 2020.

The emerging use of PMs is in photodynamic therapy. Such an application was demonstrated in recently reported antibacterial photodynamic therapy using hypocrellin A, a photosensitizer. A lipase-sensitive polymer is required for this purpose, and methoxy poly (ethylene glycol)-block-poly(ε-caprolactone) (mPEG-b-PCL) is a suitable one. This system was tested for its use against methicillin-resistant *Staphylococcus aureus* (MRSA) infections. The hypocrellin A release was lesser from micelles and resulted in higher minimum inhibitory concentrations compared to free hypocrellin A, and the in vivo performance was better for the micelles. The in vivo studies with hypocrellin-A-loaded micelles in mice showed a survival rate of 86% when the hypocrellin A dose was used at 10 mg/kg. Furthermore, no antibacterial effect was observed with hypocrellin A or hypocrellin-A-loaded micelles without the application of light (Figure 6A–C) [22].

More PMs were studied for MRSA infections. In such a study, chitosan-grafted polycaprolactone/maleic anhydride-pyrazinamide (CS-g-PCL/MA–PZA) polymer was used for the dual delivery of rifampicin (RF) and pyrazinamide (PZA). The synthesis of the polymer involved the steps of preparing chitosan-grafted polycaprolactone and maleic anhydride-pyrazinamide separately and then finally the combination of both these structures. The dialysis method was used for the preparation of rifampicin-loaded micelles. A prolonged drug release was observed from the micelles. After 12 days, 76.54% and 83.25% of rifampicin and pyrazinamide, respectively, were released from the micelles. The antibacterial effect of the formulation was promising for the drug delivery of MRSA drugs such as rifampicin and pyrazinamide (Figure 6D) [84].

When chlorophyll was delivered in the form of micelles formed from poloxamer, a significant photosensitization effect was used. This was observed as the photodynamic inactivation of *Staphylococcus aureus*. Photodynamic therapy is dependent on the micelle system by enhancing the uptake of chlorophyll by the bacterial cells [85].

### 8.4. Topical

Chitosan and soya lecithin co-polymer can be used for the preparation of micelles for drug delivery. Such a copolymer has been used for the delivery of thymoquinone for wound healing purposes. The polymer itself has the properties of dermal regeneration and tissue repair. Furthermore, the micelles enhance the solubility of thymoquinone, which has otherwise poor solubility in water. This co-polymer has a low critical micelle concentration of 6.5 μg/mL. The size of the micelles varies from 50–100 nm. An entrapment efficiency of more than 97% with an effective drug loading of around 32% was observed [35].

### 8.5. Transdermal

**Figure 7:**
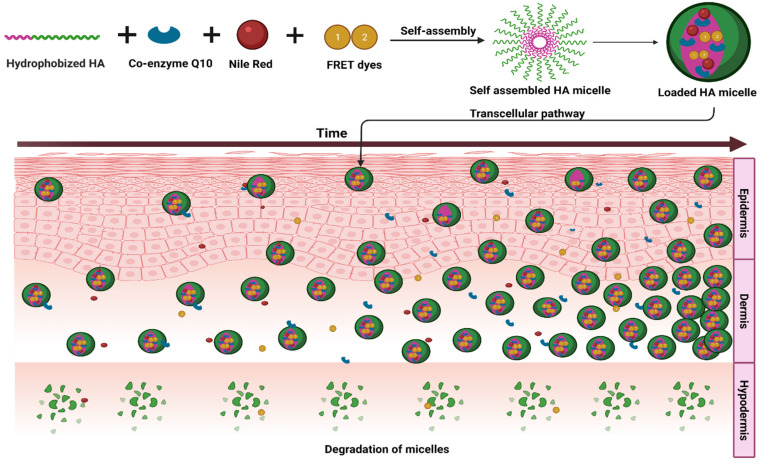
Synthesis of HA micelle and its internalization and accumulation pattern on skin layers.

Hyaluronan-based PMs were studied for transdermal delivery and proved to be effective in transdermal delivery. In fact, hyaluronan itself could act as a permeation enhancer [86]. A study carried out with nile red, coenzyme Q10 and a fluorescence resonance energy transfer probe was successful in elucidating the mechanism of drug transport across the skin. The micelles exhibited transcellular internalization followed by accumulation on the epidermis (less) and dermis (more). As the incubation time increased, the accumulation also increased. The fluorescence studies suggested a co-transport mechanism. At the same time, the fluorescence resonance energy transfer studies witnessed the destruction of polymer micelles as it reached more depths of the skin layer. The PMs were able to enhance the in vitro and in vivo performance of the coenzyme Q10. Moreover, the cream formulation prepared with it was found to be stable (Figure 7) [19]. In another study by the same group, the mechanism of uptake of hyaluronan-based PMs was studied and established. They used curcumin as the drug molecule in their study. They studied micelles of both oleyl-hyaluronan and hexyl-hyaluronan. Nile blue was used as the fluorescence tracer. Their studies suggested both active and passive mechanisms for the transport of drug carriers. In particular, oleyl-hyaluronan was found to increase the fluidity of cell membrane and enhance passive transport [20].

The anti-psoriasis effect of mycophenolic acid can be enhanced by formulating and delivering in polymer micelles. A conjugate prepared with poloxamer and mycophenolic acid is useful against psoriasis when evaluated in an in vitro model of tumor necrosis factor-α-induced HaCaT cells. The conjugate has a much better micelle-forming ability (12 times lower critical micelle concentration) than the poloxamer alone. The formation of conjugate micelles enhances water solubility and therapeutic activity. It has been further noted that micelles show an enzyme-dependent sustained-drug-release effect [31].

### 8.6. Periodontal

SP600125 is a drug that is useful against periodontal disease and is a c-Jun NH2 terminal kinase inhibitor. This drug was delivered using poly (ethylene glycol)-block-poly (ε-caprolactone) micelles. Subsequently, a micelle-in-nanofiber membrane was prepared and evaluated. The core of the core–shell nanofiber was prepared with bone morphogenetic protein-2. The system was prepared by a coaxial electrospinning method. The system showed a sequential release of SP600125 and bone morphogenetic protein-2. In vivo studies in beagle dogs demonstrated the ability of the system to reduce alveolar damage and the enhancement of bone defects [40].

In another study, PMs prepared with D-α-tocopherol PEG 1000 succinate were used for the delivery of baicalin against periodontal infections. The thin-film hydration method was used for the preparation of the baicalin-loaded PMs. The micelles provided a sustained baicalin release for up to 96 h. These drug-loaded micelles had low cytotoxicity compared to the free drugs. The efficacy of the system was demonstrated in the rat periodontitis model [87].

### 8.7. Intranasal

Micelles for the purpose of delivering rotigotine, a dopamine receptor useful against Parkinson’s disease, via the nose-to-brain route have been reported using mPEG-PLGA polymer. The PMs were prepared using the solvent evaporation method. The rotigotine-loaded micelles were 88.62 ± 1.47 nm in size and had an acceptable PDI of 0.237 ± 0.01. The statistically optimized formula was having an entrapment efficiency of 93.5 ± 0.79% and drug loading of 19.9 ± 0.60%. The PMs were further converted into a thermoresponsive gel for intranasal application. The in vitro drug releases were 75.1 and 52.9% from the micelles and gel, respectively, within 48 h. The in vivo brain-targeting efficacy of the system was studied in rats. The micelles and micelle-loaded gel were able to deliver a higher percentage of the drug to the brain compared to an intravenously administered drug solution. The micelle-loaded gel formulation was better than simple micelle dispersion most likely due to enhanced drug retention in the nasal cavity by the gel formulation. Furthermore, the gel formulation was proved to be safe for intranasal application when evaluated for ciliary movement [17].

### 8.8. Gene Delivery

PMs have been utilized for their potential in gene delivery applications, too. Polyethylenimine is a cationic polymer widely used for gene delivery applications. In a reported study, the polyethyleneimine conjugate of a hydrophobic peptide was prepared. The conjugate was amphiphilic and formed a core–shell-type micelle in an aqueous medium. The core–shell micelle formed provided a positive surface charge to the micelles, owing to the presence of cationic polyethyleneimine. This facilitated the loading of negatively charged plasmid DNA. When tested in vitro, good transfection efficiency was noted. The system has been proposed for effective gene delivery [88].

## 9. Theranostic Applications

Dye supramolecular assemblies (J-aggregates) render better harvesting of energy and therefore have enhanced theranostic utility. PMs have been found to control the J-aggregation process. The indocyanine green J-aggregates are entrapped, and a hierarchical assembly between ICG J-aggregate and the micelle polymer (phospholipid-poly(ethylene glycol)) is formed. The tumor-targeting can be achieved by an aptamer. In addition, a chemotherapeutic agent can be used. Such a system is useful for both imaging and phototherapy applications. The reported system has shown no toxicity or side effects even after 24 days of the treatment [89].

The application of the incorporation of fluorophores in PMs has been established recently. These systems possess potent uses in theranostic applications. In such a reported system, poly(ethylene glycol)-poly(hydroxyoctanoic acid) was studied after labeling with nile red and covalently rhodamine. The fluorophores displayed sufficient fluorescent stability during in vivo and in vitro testing [90].

## 10. Advanced Polymeric Micelle Systems

### 10.1. Brain-Targeted Systems

To overcome the blood–brain barrier and to deliver the drug to glioma, PMs are suggested. In a study, the dual functionalization of Pluronic^®^ P105 with glucose and folic acid was carried out for the purpose of delivering doxorubicin to glioma. The PMs were found to enhance the drug delivery to glioma and also to enhance the survival time when studied in mice [28].

Polymer prodrugs ccan provide specific advantages depending on the constituent polymer chosen for the preparation of the prodrug polymer. A redox-sensitive polymer prodrug of camptothecin can be prepared by using PEG polymer. Furthermore, iRGD, a tumor-penetrating peptide useful in drug targeting, can also be attached to form the final polymer product. Such a camptothecin-S-S-PEG-COOH-iRGD system has the self-assembling ability to form micelles. These micelles decorated with iRGD peptide have been shown to be useful in crossing the blood–brain barrier and targeting glioma. In the reported study, a photosensitizer, IR780, was also included to facilitate combination therapy. The micelles formed had a particle size of 140.68 ± 8.53 nm. The camptothecin loading efficiency was found to be 11.07%. The significant anti-glioma effect of the developed stem was established in a mouse model [91].

Magnetic PMs are another approach for brain-targeted drug delivery. A combination of a suitable polymer and super paramagnetic iron oxide nanoparticles (SPIONs) can achieve such a system. In a study, methoxy poly(ethylene glycol)-poly (caprolactone) copolymer was used as the polymer for the formation of PMs. The SPIONs prepared with the co-precipitation method were used for the preparation of naproxen-loaded magnetic PMs by the solvent evaporation technique. The particle size of such a system is important in the case of brain-targeted drug delivery; a lower particle size favors higher uptake. Particle sizes lower than 150 nm were found to be optimum for brain-targeted drug delivery. The magnetic nanoparticles supplemented brain-targeting along with the PMs for intravenous administration in rats, and thus the biodistribution of naproxen in the brain was significantly improved when delivered using the magnetic PMs. The prolongation of the circulation time was also achieved by the developed system [92].

### 10.2. Mitochondria-Targeted Systems

**Figure 8:**
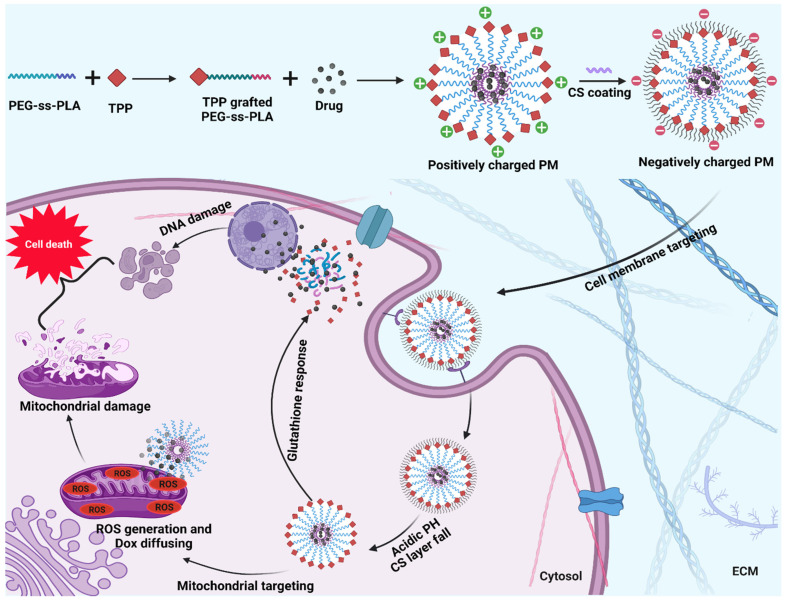
Fabrication of dual targeting and dual-responsive polymeric micelle for mitochondrial targeting.

PMs for mitochondria targeting have also been reported. In a study, doxorubicin was delivered by dual targeting and dual responsive polymeric micelle prepared by using triphenylphosphonium-grafted PEG-poly(D,L-lactide) copolymer with a disulfide linkage between poly(ethylene glycol)(PEG) and poly(D,L-lactide). A chondroitin sulfate coating was provided on the micelles to mask the positive charge of triphenylphosphonium. This coating further provided a longer circulation time and cell membrane targeting. When the system reached the lysosomes/endosomes with an acid pH, the coating of chondroitin sulfate shed off, and the triphenylphosphonium (TPP) molecules were exposed, which helped in the anchoring of the system and thus targeting mitochondria. In response to glutathione, the disulfide bond would break, and diffused doxorubicin would damage the DNA. The synergistic effects of the micelle showed greater cytotoxicity (Figure 8) [30].

### 10.3. Tumor-Targeted Systems

To target the micelles to tumor cells, folate tethering is possible. Folate decorations on dactolisib-loaded micelle surfaces have been shown to target folate-receptor-positive cells. These micelles were prepared by the self-assembly of poly(ethylene glycol)-*b*-poly(acrylic acid). In the study, the drug was conjugated to the core of the PMs by platinum (II)-based linking. The folate tethering was performed by thiol-maleimidyl coupling. The folate tethering showed lower IC50 values both in KB and A549 cell lines. The micelles without folate tethering showed lower activity. The folate-mediate uptake was confirmed by a competitive-binding study using free folate [15].

Chitosan-stearic acid micelles were used for targeting monocytes, which could be utilized for tumor-targeted drug delivery. The chitosan-stearic acid polymer was prepared using 1-ethyl-3-(3-dimethyl-aminopropyl) carbodiimide chemistry. The formation of an amide linkage resulted in the formation of the polymer. The formation of the polymer was confirmed by ^1^H NMR spectroscopy. The prepared micelles had a size of 85.93  ±  0.68 nm and zeta potential of 23.17  ±  0.90 mV. The selective uptake of the micelles by the monocytes was demonstrated in the study. Moreover, a high rate of the internalization of micelles by tumor macrophages was observed. In addition to the above-mentioned studies, in vitro release studies were also carried out with the micelles in phosphate-buffered saline (pH 7.4) after loading doxorubicin. The micelles were stable and released only less than 25% of the drug even after 72 h [16].

Ureido-modified carboxymethyl chitosan-graft-stearic acid was another polymer tried for the drug delivery purpose using PMs. The delivery of clarithromycin was tried with such a system. The polymer was prepared by the conjugation of urea to stearic-acid-modified carboxymethyl chitosan. The PMs were prepared by ultrasonication, and the formed micelles were sized around 200 nm. This clarithromycin delivery system was aimed to target *Helicobacter pylori*. The PMs were found to be stable and biocompatible. The antibacterial activity was found to be significantly higher than that of free drugs [93].

**Figure 9:**
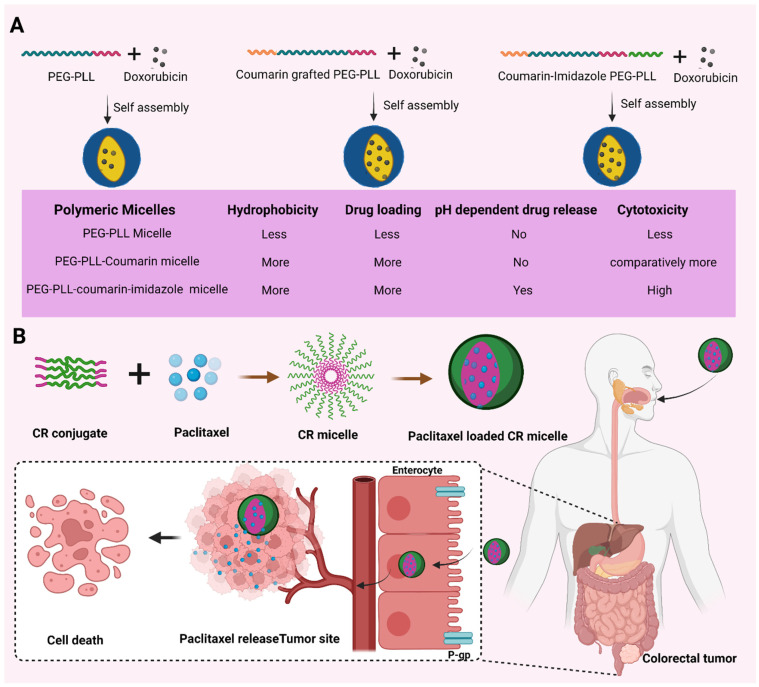
(**A**) Coumarin and imidazole grafting for enhanced drug loading and pH-dependent drug release in doxorubicin-loaded PMs. (**B**) CR-conjugate polymer synthesis and activity on the colorectal tumor.

Improving drug loading in PMs could be a possibility for their better use as a drug carrier. Such a strategy was used on pH-responsive micelles formed with methoxy poly(ethylene glycol)-block-poly(l-lysine) (mPEG-*b*-PLys) polymer. The study aimed at the use of coumarin for enhancing hydrophobicity and thus enhancing the doxorubicin loading capacity by π–π interactions. In the study, coumarin and imidazole-grafted polymer was prepared. The drug loading of micelles formed from coumarin- and imidazole-grafted polymer, coumarin alone grafted polymer and the polymer alone was compared. The coumarin- and imidazole-grafted micelles showed a high drug loading of 17.2%, whereas the coumarin alone grafted micelles showed 16.5%. These values were very high compared to the 9.6% doxorubicin loading obtained with the unmodified polymer. In addition, the imidazole-modified polymer showed a pH-dependent drug release. Furthermore, these coumarin- and imidazole-grafted micelles had rapid cellular uptake and thus better in vitro cytotoxicity (Figure 9A) [21].

The permeation of paclitaxel was found to increase both in the duodenum and colon when administered after loading into PMs. The carboxymethyl chitosan-rhein (CR) conjugate polymer was used for the purpose. This system exhibited a good drug loading capacity of 35.46 ± 1.07%. It was observed that the paclitaxel-loaded PMs could enhance permeation by bypassing the P-glycoprotein efflux. Bioimaging studies in mice have indicated that drug-loaded micelles are absorbed as a whole and reach the target tumor site. In vitro uptake studies in Caco-2 cells have also confirmed that the entire micelle gets absorbed and not the drug alone into the erythrocyte [24].

In another chitosan-based polymeric micelle for the delivery of paclitaxel, gallic acid-chitosan-D-α-tocopherol PEG 1000 succinate conjugate was used. This copolymer has the ability to enhance bioadhesion and the inhibition of p-glycoprotein efflux and drug metabolism. Furthermore, the micelles are able to enhance the solubility and bioavailability of paclitaxel. The paclitaxel-loaded micelles are able to produce significant anti-cancer activity. The anti-cancer effect has been compared with Taxol^®^ in mice [25].

In another study, paclitaxel delivery using a redox-sensitive graft polymer with a structure of mPEG-hyaluronic acid (deoxycholic acid)-*N*-acetyl-*L*-cysteine is reported. The paclitaxel was loaded during the self-assembly of the polymer in aqueous media and had an entrapment efficiency of 73.8% and a drug loading of 15.6%. The drug-loaded micelles were 147 nm in size and had a zeta potential value of −38.3 mV. Good biocompatibility, in vitro anti-cancer activity and in vivo antitumor effects were observed with the use of this system [94].

A zwitterionic polymer, *N*-deacetyl hyaluronic acid dodecylamine, was used for pH-responsive delivery of doxorubicin. This polymer changes its negative charge to positive when the pH changes from 7.4 to 6.2. This polymer is stable in serum and is less cytotoxic. Moreover, doxorubicin-loaded micelles prepared with this polymer show good cytotoxicity against MCF-7 cells. In vitro cellular uptake studies have shown that doxorubicin uptake is more when delivered as a PM than as free doxorubicin. CD44 receptor-mediated endocytosis was suggested by the study [95].

Doxorubicin in PMs formed with poloxamer has been found to be effective against triple-negative breast cancer cells. The free doxorubicin is ineffective when compared to the effect of a drug-loaded polymer. The doxorubicin-loaded PMs enhance cytotoxicity and potency [27].

After aggregation, fluorescent molecules show enhanced emission, and such a phenomenon is known as aggregation-induced emission. This phenomenon has been used to load doxorubicin in a mPEG-*g*-ε-polylysine-tetraphenylethylene polymer on self-assembly. In this system, the poly(ethylene glycol) serves the purpose of providing a hydrophilic coating layer rendering the micelles high biocompatibility and enhancing circulation time. Moreover, tetraphenylethylene provides the hydrophobic core and facilitates the aggregation-induced emission effect for doxorubicin loading. Compared to free from, doxorubicin-loaded in the micelles have been found to have higher uptake in HT-29 cells along with enhanced cytotoxicity [29].

In a study with doxorubicin-loaded micelles, methoxy poly(ethylene glycol)-*b*-poly(allyl glycidyl ether)-*b*-poly(ε-caprolactone) was used as the polymer. The polymer underwent self-assembly and facilitated doxorubicin encapsulation in the formed micelles. In particular, the interfacial crosslinking occured by thio-ene reactions [32].

In another interesting study, PMs were formed with PDEA-b-P(ABMA-co-OEGMA) polymer, with a prodrug of 6-mercaptopurine and doxorubicin attached to it. The prepared micelles were 116 ± 2 to 130 ± 2 nm in size. These micelles had the advantage of both redox-sensitive and pH-sensitive properties. The redox-sensitive effect resulted in the release of 6-mercaptopurine, whereas the pH-sensitive effect released the doxorubicin, thereby providing a fast intracellular dual-drug release effect. The acidic pH resulted in a change in zeta potential from −7.29 ± 0.76 to +9.31 ± 1.11 mV, which enhanced the internalization of the micelles and thus the cytotoxicity. The graft ratio was found to influence the cytotoxicity of the system. The micelles performed satisfactorily in HeLa cells in vitro [33].

The self-assembly of an amphiphilic polypeptide was used to deliver cytochrome C to target tumor cells by hypoxia-induced drug release. The hypoxia-responsive methoxy PEG-block-poly (diethylenetriamine-4-nitrobenzyl chloroformate)-l-glutamate was used as the amphiphilic polymer. The conjugation of 4-nitrobenzyl chloroformate to the system made it a hypoxia-responsive polymer. The micelles were able to show effective uptake by HepG2 liver cancer cells. Under hypoxic conditions, the cytochrome c-loaded micelles were shown to show good cytotoxicity in HepG2 cells [34].

Poly(aspartic acid) derivatives with phenyl borate serine side groups could be used as reactive-oxygen-species-sensitive polymers. Here, the phenyl borate serine group acted as the reactive-oxygen-species-sensitive part. The doxorubicin-loaded micelles were found to have desirable hydrogen peroxide-dependent drug release. Doxorubicin was effectively delivered using such a system and had a high selectivity toward cancer cells. In the study, cytotoxicity was more in A549 cells than L929 cells as a result of higher doxorubicin delivery to A549 cells [36].

In a special type of drug delivery application, PMs were loaded into a nanofiber patch prepared using psyllium husk mucilage. Cholic-acid-conjugated poly (bis (carboxyphenoxy) phosphazene) was used as the polymer for paclitaxel-loaded micelle preparation. The nanofibers prepared by coaxial electrospinning using psyllium husk mucilage had stimulus-sensitive core–shell structures. The system showed good transport across the epidermis. Compared to free paclitaxel, the micelle-loaded nanofibers patch showed higher cytotoxicity in MCF-7 cells [37].

When the polymer poly (α-azide caprolactone-co-caprolactone)-b-poly (2-methacryloyloxyethyl phosphorylcholine) is conjugated to 7-ethyl-10-hydroxycamptothecin, a polymer prodrug is obtained. This polymer with a zwitterionic nature forms micelles and is used for the dual delivery of doxorubicin and 7-ethyl-10-hydroxycamptothecin. The dual-drug-loaded micelles show better uptake by 4T1 cells. This system has the ability to overcome dose-limiting toxicity and drug resistance in cancer chemotherapy [39].

Lipid and polymer dual-grafted system of chlorin e6 forms micelles. This micelle-forming amphiphilic polymer has been studied for photodynamic therapy. An amphiphilic polymer is obtained when chlorin e6 is conjugated to PEG and dioleoyl phosphatidyl ethanolamine with a structure of PEG- chlorin e6- DOPE. The developed system shows enhanced phototoxicity compared to chlorin e6 [96].

PEG and poly(ω-pentadecalactone-co-*N*-methyldiethyleneamine sebacate-co-2,2′-thiodiethylene sebacate) can show both pH and reactive oxygen species sensitiveness. These properties are useful in tumor-targeted drug delivery and have been demonstrated with docetaxel. In this study, the drug-loaded PMs were prepared using a self-assembly method and were further purified by dialysis. The size of the micelles ranged from 44 to 67 nm. The anti-tumor activity was dependent on the percent of thiodiethylene sebacate group in the polymer, and the micelle formulations performed better than a marketed formulation of docetaxel [97].

**Figure 10:**
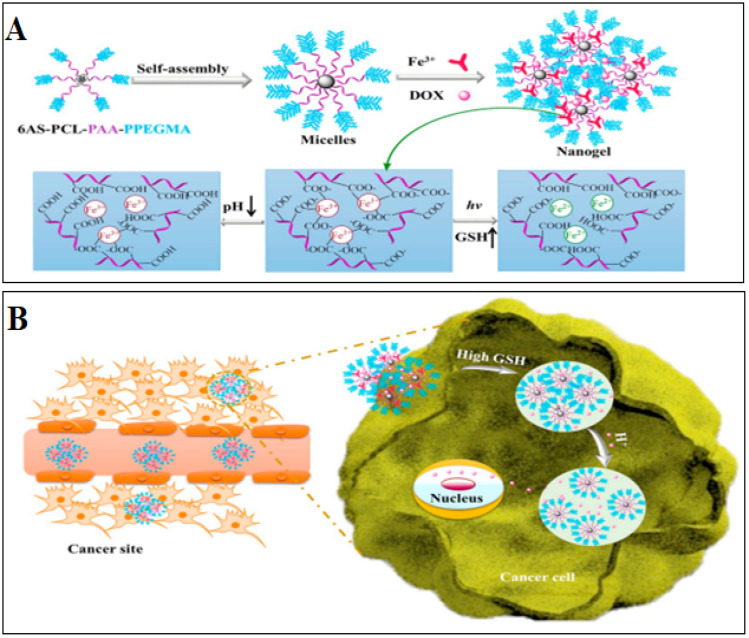
(**A**) Schematic representation of micelle formation, gelation with Fe^3+^ and pH-, UV- and GSH-triggered drug release mechanism. (**B**) Intracellular release of multi-responsive nanogels encapsulated with anticancer drugs.

A six-arm star-shaped amphiphilic copolymer was recently reported for the delivery of the anti-cancer drug doxorubicin. The system was responsive to pH, redox and UV irradiation, thus presenting a multi-stimuli responsive drug delivery system. Poly (caprolactone)-b-poly (acrylic acid)-b-poly (poly (ethylene glycol) methyl ether methacrylate) was the polymer used for the purpose. The polymer formed micelles with a particle size of 80–110 nm and zeta potential of −30.19 mV. The micelles formed hydrogel by complexation with the addition of Fe^3+^. An increase in size was observed after the complexation with Fe^3+^ to 150–200 nm. The drug was entrapped in the hydrogel by addition during the cross-linking process with Fe^3+^. The in vitro drug release was higher in the acid medium. In addition, simulated redox and UV irradiation testing also showed high doxorubicin release. In addition, the carrier hydrogel was found to be non-toxic against HepG2 cells (Figure 10) [98].

In another triple-stimuli responsive tumor-targeted system, a graft polymer PSNC-*g*-mPEG/TPE was used. This amphiphilic co-polymer self-assembled in aqueous solutions and entrapped the drugs. The system was responsive to pH, ROS and enzymes, thus providing an option for tumor-targeted drug delivery. Nile red was used as the model drug and probe in the study. The prepared micelles were below 200 nm in size. The in vitro cytotoxicity studies in U87, HL-7702 and RAW264.7 cells confirmed the biocompatibility of the carrier system [99].

Poly(polyethylene glycol methyl ether acrylate) forms brush-shaped polymers with an amphiphilic nature. Reversible addition-fragmentation chain transfer polymerization was used for the preparation of the polymer. Ruthenium (II) complex was used as the anti-cancer agent in the study. A thin-film hydration method followed by dialysis was employed for the preparation of drug-loaded micelles. The average particle size reported for the micelles was 21 nm. The drug loading was around 1.5% *w*/*w*. The poor water solubility of the drug was slightly improved by the formulation as micelles. Although the micelles showed no toxicity, the cytotoxicity of the ruthenium (II) complex was significantly increased, both in the absence and presence of light [100].

PMs prepared with poly[N-isopropylacryamide-co-allyl poly(ethylene glycol)]-b-poly(γ-benzyl-l-glutamate) (P(NIPAM-*co*-APEG)-*b*-PBLG) can control drug release. Such a system was reported with doxorubicin. In the study, hydrazone bonds were utilized for attaching the drug to the core of the micelles. The system demonstrated high drug loading and entrapment efficiency of 15% and 80%, respectively. The PMs showed both pH- and temperature-responsive release of doxorubicin from the system. The doxorubicin-loaded micelles showed good cytotoxicity against Hela and 3T3 cells [101].

Ferroptosis has been identified as a method to avoid multidrug resistance in cancer chemotherapy. Ferroptosis reduces the number of persister cancer cells responsible for drug resistance in chemotherapy. In a reported study, RSL3, a ferroptotic inducer, was loaded into PMs in order to target glutathione peroxidase 4. A 30-fold increase in the activity was shown by the drug-loaded micelles compared to the control micelles when tested in drug-resistant cancer cells. The potency of the system was confirmed through an in vitro soft agar colony-forming assay. Moreover, the in vivo efficacy of the system was demonstrated in tumor-bearing mice [102].

The delivery of melphalan was tried and found promising for retinoblastoma using PMs. *N*-acetylheparosan-cystamine-vitamin E succinate co-polymer was used in the study. This polymer was biodegradable and reduction-responsive. The obtained micelles had superior serum stability and cytotoxicity. The drug-loaded micelles showed high uptake by retinoblastoma cells. Furthermore, the rapid disassembly of the micelles occured in tumor cells, thereby releasing the drug and causing cytotoxicity [103].

Matrix metalloproteinase 2–sensitive systems are ideal for tumor-targeted drug delivery. An ideal polymer for such a purpose is PEG–MMP2-cleavable peptide–phosphatidylethanolamine. This polymer can inhibit drug efflux. The polymer without the linkage of cleavable peptide attaches to the tumor cell surface at high concentrations and can reduce the internalization. However, the studied system does not have this issue due to the presence of the MMP2-cleavable peptide. A thin-film hydration method was used for the preparation of dasatinib-loaded PMs. The delivery of dasatinib in PMs resulted in a significant anticancer effect when tested in 4T1 tumor-bearing mice [104].

Zinc (II) phthalocyanine is a photosensitizer useful for cancer therapy. This photosensitizer was encapsulated in folate functionalized methoxy poly(ethylene oxide)-b-poly(L-lactide) micelles during its preparation by the dialysis method. The drug-loaded micelles were below 150 nm in size. The drug placed itself in the core of the micelles, whereas the folate groups were presented in the outer hydrophilic layer. The drug-loaded micelles showed higher cellular uptake. Furthermore, the degree of folate-functionalization favored cellular uptake. The drug-loaded micelles generated reactive oxygen species, caused damage to mitochondria and arrested the cell cycle at the G2/M phase [105].

In a liver tumor-targeted dual drug delivery system, glycyrrhetinic acid and doxorubicin were delivered using PMs. In the study, a PEG derivative of glycyrrhetinic acid (PEG-Fmoc-GA), a prodrug, was used for the preparation of PMs by the thin-film hydration method. Doxorubicin was encapsulated during the micelle formation. The study was carried out at different carrier/drug molar ratios, and the particle size ranged around 180–250 nm. The combination therapy using PMs was found to be effective and promising both in vitro and in vivo. For in vivo studies in a human liver cancer xenograft mice model, enhanced antitumor effects were noted. The micelles showed longer circulation time and accumulation in the tumor site [106]. The co-delivery of drugs had to overcome some hurdles, such as poor miscibility of the drugs and the low drug loading of the nanocarriers [107].

Dual drug delivery of anticancer agents was achieved using poly(2-methyl-2-oxazoline-*block*-2-butyl-2-oxazoline-*block*-2-methyl-2-oxazoline). The concurrent delivery of paclitaxel and cisplatin was performed using such a system. A thin-film method was used for the preparation of PMs with dual drug loading. The drug loading was more than 50%, and the co-loading was observed to cause a slow drug release in the serum. The micelles showed better tumor penetration and distribution. The anti-tumor effect was better than that of single-drug-loaded micelles. The system was predicted to be a better therapeutic option for ovarian and breast cancer [107].

## 11. Mixed Micelle Systems

Mixed polymeric micelle systems can be considered advantageous for many reasons. Some of the mixed micelle systems are already explained in the previous sections.

Mixed PMs can increase the oral bioavailability of drugs. These have been proved to improve the oral bioavailability of syringic acid. The micelles were prepared with D-α-tocopherol PEG 1000 succinate, Pluronic^®^ F127 and Pluronic^®^ F68. In addition, the syringic acid was found to have enhanced hepatoprotective action when loaded in the micelles. The micelles were found to enhance cellular internalization and delay syringic acid elimination [108].

In another mixed micelle system, PEG-poly(aspartic acid benzyl ester-random-aspartic acid hydrazine amide) conjugated to epirubicin and maleimide-PEG-poly(glutamic acid benzyl ester) was used for the preparation of micelles. The micelles were then decorated with a monoclonal antibody. The immunomicelles were very effective in targeting tumor cells [109].

## 12. Other Special Types

Magnetic micelles can be prepared using polystyrene-*b*-poly(acrylic acid)-*b*-poly(ethylene glycol) polymer and FeCl_3_·6H_2_O. The FeCl_3_·6H_2_O provides the magnetic property, and the polymer forms the micelles. As mentioned previously, in this case as well, the poly(ethylene glycol) outer layer provides hydrophilicity for the system. In these micelles, polystyrene forms the hydrophobic core. Moreover, poly(acrylic acid) facilitates electrostatic interactions with iron ions. Nile red is encapsulated in the core of the micelles. This system was proposed to be useful for drug delivery applications [110].

Unimolecular micelles are another advanced form of PMs. Such a system is reported with β-cyclodextrin-poly(acrylic acid)-paclitaxel. It is a star-shaped polymer-forming unimolecular micelle. It forms an amphiphilic system and shows a drug loading of around 59%. The cyclodextrin forms the core of the system, and the in vivo performance of the drug-loaded micelles depends on the molecular weight of the micelles. High molecular weight was found to favor higher accumulation in tumors when tested in tumor-induced mice [111].

Acid-activatable polymeric curcumin has been proposed to deliver certain specific advantages, such as site-specific drug delivery. This involves the incorporation of curcumin into the hydrophobic backbone of poly(β-amino ester) polymer. The resultant prodrug form of curcumin has been presumed to have specific use against inflammatory diseases, owing to its acid-responsive nature. Such a system can be used in therapeutic, diagnostic or theranostic applications. This system has another advantage of high drug payload compared to liposomes, nanoparticles, etc., where the drug loading is usually limited to low values. The micelles are prepared by a solvent evaporation method. The antioxidant and anti-inflammatory activities of the acid-activatable polymeric curcumin are better than free curcumin. Enhancement in the solubility of curcumin can be the reason for this advantage. The therapeutic potential of the system was observed in a mouse model of osteoarthritis [112].

A percolated micelle network can form hydrogels and can be used for drug delivery. The polymer poly(*D*,*L*-lactic acid-co-glycolic acid)-b-poly(ethylene glycol)-b-poly(*D*,*L*-lactic acid-co-glycolic acid) forms such a system. This polymer forms a thermo-responsive hydrogel. This system was demonstrated to be useful in the therapeutic application of gemcitabine in cancer chemoradiotherapy. Gemcitabine has both anti-cancer and radiosensitization effects. In the reported study, the palmityl derivative of gemcitabine was used. It is of particular interest to note that this palmityl derivative of gemcitabine, which could be considered a polymer prodrug, formed the micelles in the hydrogel. These micelles and aggregates showed a sustained-release effect. In addition, the rapid internalization of the micelles occured in 4T1 tumor cells when tested in vitro. The in vivo studies in the tumor-induced mouse model showed that a single dose of the micelle-containing hydrogel plus three-times X-ray exposure produced the tumor suppression [113].

Polymeric micelles formed of nucleic acid-synthetic polymer conjugates are an advancement in the field of drug delivery applications. These have a property of self-assembling to form micelles. These micelles have been proposed to be capable of forming super aggregates. A click-chemistry has been used in the preparation of such a reported system. Hydrophobic interactions are involved in the formation of such nucleic acid-synthetic polymer conjugates. The nucleic acid strands are placed at the surface of the micelles, rendering high biocompatibility, less toxicity and enhanced cellular uptake [114].

**Figure 11:**
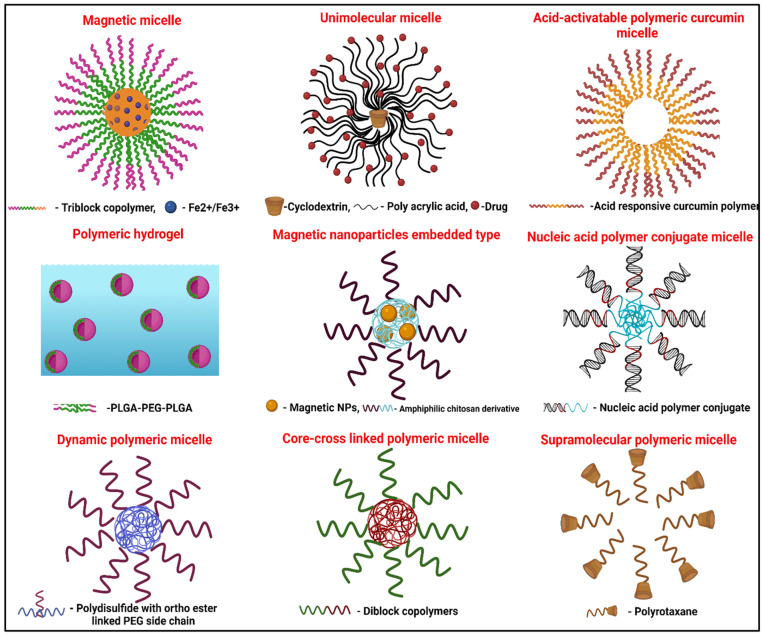
Diagrammatic representation of special types of PMs.

PMs with magnetic nanoparticles embedded present another opportunity for tumor-targeted drug delivery (Figure 11). When amphipathic chitosan polymer and Fe_3_O_4_ magnetic nanoparticles are combined, a pH-responsive drug delivery system is obtained. Such a carrier was evaluated for the tumor-targeted delivery of paclitaxel. N-(2-hydroxy) propyl-3-trimethyl ammonium chitosan chloride and alkylated PEG N-(2-hydroxy) propyl-3-trimethyl ammonium chitosan chloride polymers were studied for the purpose. The CMC of the polymer was found to be dependent on the chain length of the grafted alkyl group and the degree of substitution. Spherical and superparamagnetic nanoparticles were formed. Moreover, the system showed pH-dependent paclitaxel release [115].

A sequentially dynamic polymeric micelle with detachable PEGylation was constructed for enhanced cancer therapy. Doxorubicin encapsulated in dynamic PMs were made up of ortho ester-linked PEGylated poly(disulfide). The micelles were stable in sodium dodecyl sulfate solution at pH 7.4, but it underwent dePEGylation and dynamic size changes at tumor extracellular pH (6.4) due to the hydrolysis of the ortho ester linkage. The cleavage of the poly sulfide backbone allowed the faster release of the drug. The dynamic PMs showed stable blood circulation, better cellular uptake and cytotoxicity [116].

Supramolecular PMs are another modification reported for drug delivery purposes. In the study, nanoparticles of β-carotene were prepared using poloxamer. Supramolecular PMs are also prepared by polyrotaxane compounds formed from cyclodextrins. The nanoparticles based on cyclodextrin enhanced the cytotoxicity of melanoma cells by enhancing the water solubility of carotene [117].

Core cross-linked PMs are another type of PMs exhibiting high stability in in vivo performances. Hence, core cross-linking is an important technique for enhancing stability. These are core–shell structures formed from amphiphilic block copolymers, and the hydrophobic core part is stabilized physically or chemically. Chemical cross-linking is possible with the use of cross linkers, radical polymerization, disulfide linkage, etc., and core cross-linked PMs offer the chemical cross-linking of drug molecules for tailored release kinetics [118].

## 13. In Vivo Stability of Polymeric Micelles

PMs for intravenous injections have been reported in many studies [119,120]. Nonetheless, the reports on the evaluation of in vivo stability of PMs are fewer. The performance of the micelles as drug delivery carriers is hugely dependent on their stability in body fluids. This is more important in the case of micelles for parenteral administration. Some of the earlier studies have addressed such issues [121,122].

In vitro testing in serum is frequently employed for the prediction of in vivo stability [16]. In a recent interesting study, the in vivo stability of PEG-block-poly(ε-caprolactone) is described [123]. They used a Förster resonance energy transfer method in their study. Interestingly, their study showed that around 60% of the micelles were intact even after 72 h when administered intravenously. The in vivo stability studies were carried out in female ICR mice.

In another advanced study, the density ultracentrifugation method was used in addition to Förster-resonance-energy-transfer-based fluorescent spectrometry to evaluate the stability of PMs in body fluids. Poly(ethylene glycol-block-caprolactone) and poly(ethylene glycol-block-lactide) were used to study the stability of micelles. Serum was found to affect the stability of PMs by causing rapid dissolution. Moreover, micelles showed good stability in cerebrospinal fluid. The instability was not dependent on the dilution of the micelles on administration, as instability was shown by concentrations well above the CMC. Thus, CMC could not be considered a parameter for stability in biological fluids. Nevertheless, as expected, it was observed that biological molecules such as proteins could affect the stability of PMs in biological fluids [124].

Pluronic^®^ F127 micelles are used for loading Zileuton™ for cancer therapy. It was found that these Zileuton™-loaded micelles were able to decrease the circulating breast-cancer cells. Moreover, they were effective against tumor stem cells. This strategy could be considered important in the recurrence and spread of tumors [23].

The in vivo fate of oleyl hyaluronan PMs was studied and reported. The doxorubicin-loaded PMs, prepared by a solvent evaporation method, were found to show rapid dissociation upon in vivo administration. Even then, the doxorubicin administered encapsulated in micelles showed prolonged circulation time. The elimination of the micelle polymer was complete by 72 h. Furthermore, it was noted that the mechanism of elimination differed for oleyl hyaluronan and hyaluronan [125].

## 14. Patents on Polymeric Micelles

The flexibility of PM formulations renders them sufficiently adaptable for modifications to suit specific applications. With the versatility in the method of preparation of PMs and the availability of a variety of polymers for the fabrication of PMs, there exist a plethora of opportunities for novel and inventive strategies related to PMs and their applications in drug delivery. The recent patent publications related to PMs and their drug delivery applications are provided in Table 2.

## 15. Clinical Applications of Polymeric Micelles

Based on the versatility of the PMs, their prospects in clinical applications are promising. Estrasorb (Novavax, Inc., Gaithersburg, AR, USA) is an FDA-approved PM system for the controlled delivery of estradiol hemihydrates and is approved for clinical use by USFDA [126]. SP1049C (Supratek Pharma), a doxorubicin-loaded Pluronic^®^-based PMs was approved for orphan drug status for gastric cancer. Moreover, some are under or have completed clinical trials (Table 3) and can be expected to obtain regulatory approval soon for clinical use. It can be seen that PMs of paclitaxel (Genexol-PM^®^) are the most clinically studied formulation. Interestingly, most of the clinical trials with PMs are related to cancer chemotherapy. The enhanced permeability and retention effect of PMs significantly contributes to this advantage. Furthermore, PMs are also useful for both active and passive targeting [127].

## 16. Conclusions and Perspectives

Drug delivery using PMs has witnessed unparalleled advances recently. The type of PMs formed is mainly influenced by the type of polymers used and intermolecular forces. Moreover, the method of their preparation depends on the physicochemical characteristics of the block copolymers, which in turn significantly affect the drug encapsulation efficiency. CMC determination and morphological and physicochemical characterizations are important for the understanding of the type and structure of PMs. Interestingly, the molecular dynamics of PMs have been useful in studying drug loading. For drug delivery applications, the safety, quality and stability of PMs are other important aspects to consider.

The enhancement of drug bioavailability is one of the advantages of PMs for oral administration. Parenteral delivery using PMs provides efficient and targeted drug delivery. PMs are also useful for photodynamic therapy and antibacterial drug delivery. Recent studies have exploited the advantages of PMs for topical, transdermal and periodontal drug deliveries. Similar to the oral route, PMs have shown enhanced drug bioavailability by intranasal route also. Moreover, the presence and the versatility of polymers provide an immense contribution to the gene delivery and theranostic applications of PMs. Notably, some advanced polymeric systems have been recently reported, such as brain-, mitochondria- and tumor-targeted PMs. Among these, tumor-targeted systems of PMs are extensively reported, recently owing to the importance of their application. Mixed micelles and other special types of PM have emerged with special features. PMs capable of forming super aggregates are such an example.

Even though in vitro stability studies of PMs are available, in vivo studies are limited. Nevertheless, some patented PMs are useful for drug-delivery applications. Moreover, some PMs are under clinical trials for their use. The flexibility of PM formulations provides great hope and openings for future research on their drug delivery applications. It is presumed that PMs can make significant contributions to future oral drug-delivery systems. Presently, studies on PMs are more focused on anti-cancer therapies. However, the expansion of the applications of PMs in the therapy of other diseases can be successful. Ophthalmic and transdermal delivery are other areas that can benefit from PMs in the future. Drastic advancements in nucleic acid and protein deliveries can also be achieved using PMs. Thus, PMs are under extensive studies and can prove to be useful tools to overcome many unresolved issues in drug delivery.

## Figures and Tables

**Table 1:** Different types of polymers used for micelle fabrication and their methods of preparation.

Sl. No.	Polymer	Method of Preparation	Drug	Reference
1	Block copolymer of mPEG (5 kDa)-PCL (2 kDa)	Continuous processing	Curcumin	[14]
2	Poly(ethylene glycol)-*b*-poly(acrylic acid)	Self-assembly	Dactolisib	[15]
3	Chitosan-stearic acid	Self-assembly	Doxorubicin	[16]
4	mPEG-PLGA	Solvent evaporation method	Rotigotine	[17]
5	disteraroylphosphatidylethanolamine-(PEG)	Solvent evaporation method	Paclitaxel	[18]
6	Hyaluronan	Solvent evaporation method	Coenzyme Q10	[19]
7	Hexyl-hyaluronan and oleyl-hyaluronan	Solvent evaporation method	Curcumin	[20]
8	Coumarin and imidazole grafted poly(ethylene glycol)-b-poly(l-lysine)	Mixing and dialysis Nanoprecipitation	Doxorubicin	[21]
9	mPEG poly(ethylene glycol)-*block*-poly(ε-caprolactone) (PEG-*b*-PCL)	Thin-film hydration method	Hypocrellin A (photosensitizer)	[22]
10	Pluronic^®^ F127	Thin-film hydration technique	Zileuton^TM^	[23]
11	Carboxymethyl chitosan-rhein conjugate	Self-assembly	Paclitaxel	[24]
12	Gallic acid-Chitosan-D-α-tocopherol PEG 1000 succinate	Ultrasonic emulsification	Paclitaxel	[25]
13	D-α-tocopherol PEG 1000 succinate (TPGS)	Thin-film dispersion	Laquinimod	[26]
14	Poloxamer	Not mentioned	Doxorubicin	[27]
15	Functionalized poloxamer	Not mentioned	Doxorubicin	[28]
16	Methoxypoly(ethylene glycol)-*graft*-ε-polylysine-tetraphenylethylene	Self-assembly	Doxorubicin	[29]
17	triphenylphosphonium (TPP) grafted poly(ethylene glycol)(PEG)-poly(*D*,*L*-lactide)(PLA)	Solvent evaporation method	Doxorubicin	[30]
18	Poloxamer	Self-assembly	Mycophenolic acid	[31]
19	Methoxy poly(ethylene glycol)-*b*-poly(allyl glycidyl ether)-*b*-poly(ε-caprolactone)	Solvent evaporation method	Doxorubicin	[32]
20	PDEA-b-P(ABMA-co-OEGMA)	Sonication	6-mercaptopurine and doxorubicin	[33]
21	mPEG-b-P(Deta-NBCF)LG	Self-assembly	Cytochrome C	[34]
22	Chitosan-lecithin	Sonication	Thymoquinone	[35]
23	Poly(aspartic acid) derivatives with phenylborate serine side groups	Dialysis	Doxorubicin	[36]
24	Cholic acid conjugated poly (bis (carboxyphenoxy) phosphazene)	Ultrasonication	Paclitaxel	[37]
25	Poly-(lactic-co-glycolic)-b-poly (ethylene glycol)-maleimide	Dialysis method	Nilotinib	[38]
26	Poly (α-azide caprolactone-co-caprolactone)-b-poly (2-methacryloyloxyethyl phosphorylcholine)	Dialysis method	Doxorubicin and 7-Ethyl-10-hydroxycamptothecin	[39]
27	Poly (ethylene glycol)-block-poly (ε-caprolactone)	Thin-film hydration	SP600125 and Bone Morphogenetic Protein-2	[40]

**Table 2:** Recent patents related to PMs and their drug delivery applications.

Sl. No.	Publication No. (Year)	Title	Summary of Invention
1.	WO/2020/228265 (2020)	“Pharmaceutical compositions containing mixed PMs”	Describes drug-loaded 1–1000 nm-sized mixed PMs.
2.	111686261 (2020)	“Adriamycin-polymeric micelles-nucleophosmin-binding protein (ADR-PMs-NMBP) with anti-acute lymphoblastic leukemia (ALL) activity and preparation method of ADR-PMs-NMBP”	Describes the preparation method of the ADR-PMs-NMBP.
3.	111686076 (2020)	“Doxorubicin-loaded polymeric micelle as well as preparation method and application thereof”	Describes the preparation and applications of DOX-loaded PMs. Two amphipathic PMs were described for the purpose.
4.	201811043353 (2020)	“Improved wound healing topical composition of thymoquinone”	Thymoquinone-loaded PMs are prepared using chitosan and soy lecithin for wound healing.
5.	CN111658783A (2020)	“Switch type glucose-responsive double-layer cross-linked polymer micelle drug delivery system and preparation method and application thereof”	Glucose-responsive double-layered cross-linked PM.
6.	CN111978520A (2020)	“PEG monomethyl ether-polylactic acid segmented copolymer, polymer micelle medicine and preparation method”	Preparation of PMs using PEG monomethyl ether-polylactic acid segmented copolymer for medical application.
7.	CN111978553A (2020)	“Triple-stimulus responsive interfacial crosslinked polymer micelle and preparation method and application thereof”	Triple stimulus-responsive hydrophobic polymer-based PMs. Enhanced drug loading and rapid drug release to stimulus.
8.	CN111330014A (2020)	“Acid-responsive cross-linked polymer prodrug and preparation method and application thereof”	Includes a vinyl alkyl ether acrylate monomer. Provides high drug loading for hydroxyl-group-containing drugs.
9.	CN111888357A (2020)	“Chemotherapy drug-co-loaded crizotinib prodrug polymer micelle and preparation method thereof”	PM is prepared by self-assembling an amphiphilic diblock copolymer and hydrophobic crizotinib.
10.	CN111214438A (2020)	“Method for preparing doxorubicin-loaded polymer micelles with different sizes”	Size controlled doxorubicin-loaded PMs using a micro-reaction system.
11.	United States Patent 10,799,455 (2020)	“Micelles containing alpha-lipoic acid as a transdermal drug delivery system”	PMs with alpha-lipoic acid and vinpocetine for transdermal delivery.
12.	109966508 (2019)	“pH-sensitive targeting polymeric micelles PPi-Far-PMs, and preparation method and application thereof”	PMs for the release of farnesol to the decayed tooth by targeting the pyrophosphate and acid environment.
13.	WO/2019/129657 (2019)	“Actively targeted polymeric micelles for drug and gene delivery”	PMs prepared of an amphiphilic block copolymer of polyoxyethylene and polyoxypropylene blocks.
14.	CN108524933 (2018)	“Carrier capable of inhibiting tumor multidrug resistance and preparation method thereof”	PMs are prepared by targeting molecule-PEG-pyrene amphiphilic polymer system.

**Table 3:** Present clinical trials listed in the clinical trial registry (ClinicalTrials.gov) on PM-based drug delivery systems.

Sl. No.	ClinicalTrials.gov Identifier	Details	Phase (Status)
1.	NCT00912639	Cremophor EL-free PMs of paclitaxel (Genexol-PM^®^) for taxane-pretreated recurrent breast cancer	Phase 4 (Not available)
2.	NCT01023347	A comparative trial between two strategies; Paclitaxel (Genexol^®^) and cisplatin vs. Genexol-PM^®^ and cisplatin, in non-small cell lung cancer	Phase 2 (Completed)
3.	NCT00886717	Paclitaxel-loaded PMs in advanced ovarian cancer	Phase 2 (Not available)
4.	NCT03585673	Docetaxel-loaded PMs in esophagus squamous cell carcinoma	Phase 2 (Recruiting)
5.	NCT01426126	Genexol-PM^®^ in advanced urothelial cancer earlier treated with gemcitabine and platinum	Phase 2 (Completed)
6.	NCT02639858	Docetaxel-loaded PMs in recurrent or metastatic head and neck squamous cell carcinoma	Phase 2 (Not available)
7.	NCT00111904	Paclitaxel-loaded PMs in unresectable locally advanced or metastatic pancreatic cancer	Phase 2 (Completed)
8.	NCT01770795	Genexol-PM^®^ in untreated metastatic non-small cell lung cancer patients	Phase 2 (Completed)
9.	NCT02739529	Genexol-PM^®^ in gynecologic cancer	Phase 1 (Not available)
10.	NCT00882973	Genexol-PM^®^ in advanced pancreatic cancer	Phase 1 (Completed)

## Data Availability

All the relevant data are included in the manuscript.

## Abbreviations

Polymeric micelles: PMs; Dynamic light scattering: DLS; Critical Micelle Concentration: CMC; poly(ethylene glycol)-block-poly(ε-caprolactone): (PEG-b-PCL); poly (methyl methacrylate): PMMA; Atomic Force Microscopy: AFM; Scanning Electron Microscopy: SEM; Transmission Electron Microscopy: TEM; Cryogenic-Transmission Electron Microscopy: cryo-TEM; Fourier Transform Infrared Spectroscopy: FTIR; Nuclear Magnetic Resonance Spectroscopy: NMR; Small-angle Neutron Scattering: SANS; Small-Angle X-ray Scattering: SAXS; Liquid Cell Transmission Electron Microscopy: LC-TEM; Anomalous Small-Angle X-ray Scattering: ASAXS; Differential Scanning Colorimetry: DSC; Attractive polymeric micelles: APMs.
